# A multidimensional tool to measure farm stressors: development and initial validation of the farmer stress assessment tool (FSAT)

**DOI:** 10.1186/s40359-024-01929-w

**Published:** 2024-08-12

**Authors:** R. J. Purc-Stephenson, S. Dedrick, D. Hood

**Affiliations:** Department of Social Science, Augustana Faculty, University of Alberta, Camrose, AB T4V 1R3 Canada

**Keywords:** Mental health, Agricultural safety, Depression, Anxiety, Resilience, Burnout, Stress

## Abstract

**Background:**

Farming is a stressful occupation, and a growing body of research shows that farm stressors are associated with poor mental health. To date, there are few methodologically sound surveys that assess farm stressors, and none have been validated for the Canadian context. Our study aimed to: (a) investigate the types of stressors experienced by farmers, (b) develop a farm stress assessment tool and test its factor structure and internal consistency, and (c) assess its criterion-related validity to self-reported levels of anxiety, depression, burnout, and resilience among farmers.

**Methods:**

We developed a 20-item survey based on a review of the literature, examining existing farm stress surveys, and consulting 10 farmers and agricultural industry experts. Then, a convenience sample of farmers living in Alberta, Canada (Sample 1, *N* = 354) completed a questionnaire containing the 20-item farm stress survey and four validated measures that assessed depression, anxiety, burnout, and resilience. Sample 1 was used to assess the factor structure using exploratory factor analysis (EFA), internal consistency, and criterion-validity of the survey. Next, a convenience sample of farmers living outside of Alberta (Sample 2, *N* = 138) was used to evaluate the factor structure of the survey using confirmatory factor analysis (CFA).

**Results:**

The results of the EFA revealed five underlying dimensions of farm stressors: Unexpected work disruptions, Agricultural hazards, Farm and financial planning, Isolation, and Regulations and public pressure. The subscales accounted for 61.6% of the variance, and the internal consistency (Cronbach’s alpha) ranged from 0.66 to.75. Subscale correlations were below 0.44, indicating evidence of discriminant validity. Correlations between the five subscales and the four mental health outcome variables supported the criterion-related validity of the survey. The results of the CFA indicated that the data fit the model, and fit was further improved by correlating one pair of error terms.

**Conclusions:**

Preliminary analysis of our Farmer Stress Assessment Tool (FSAT) suggests it is a reliable and valid instrument for measuring a range of stressors farmers face. Implications for policy and community-based mental health interventions that help farmers manage the enduring stressors of agriculture is discussed.

**Supplementary Information:**

The online version contains supplementary material available at 10.1186/s40359-024-01929-w.

## Background

Agriculture was recognized as one of the most stressful occupations [1, 2], with chronic stress adversely affecting farmers’ mental health and well-being [3, 4]. The fourth European Working Conditions Survey, which collected data from 31 European countries, found that 32% of agricultural and fishery workers reported that work-related stress negatively impacted their mental health, a higher percentage than reported in other occupational groups [5]. Studies in Canada and the USA also reported that farming stressors significantly affected farmers’ mental health, contributing to conditions such as depression, anxiety, and burnout [2–4]. Understanding the factors contributing to the high stress among farmers is essential to inform policies and interventions aimed at enhancing farmers’ mental health. Our goal was to investigate the stressors farmers face, develop an assessment tool to measure these stressors, and evaluate its factorial structure, internal consistency, and criterion validity. First, we summarize existing farm stressor surveys, review research on the mental health of farmers, and present a theoretical framework to help understand why farm stressors may place farmers at risk for poor mental health.

Previous research has identified an array of stressors prevalent in the lives of farmers, including long work hours, time constraints, fluctuating market prices for crops and livestock, and exposure to volatile weather conditions such as droughts, floods, and wildfires [6, 7]. Efforts to measure farm stressors began in the 1990s. First, Eberhard and Poonyan [8] developed a 28-item farm stressor survey by interviewing six farmers about work-related concerns. They surveyed 362 U.S. farmers and conducted a principal components analysis that revealed six stress factors: hazardous working conditions, geographic isolation, personal finances, time pressure, weather conditions, and general economic conditions. The results showed that stress related to personal finances and time pressure was significantly related to lower life satisfaction, emotional strain, and illness frequency, while geographic isolation was associated with emotional strain and illness frequency. However, the measures used for life satisfaction and emotional strain were not validated, and the survey did not include items about government.

Next, Deary et al. [9] developed the 27-item Edinburgh Farming Stress Inventory (EFSI) by adapting previous research for farmers in the UK. The survey included stress factors such as bureaucracy, finances, isolation, uncontrollable events, occupational hazards, and time pressure. They surveyed 318 UK farmers, and the results showed that bureaucracy was the top stressor, followed by weather, time pressure, and financial concerns. A principal components analysis identified six stress factors: bureaucracy, finances, isolation, uncontrollable events, personal hazards, and time pressure. However, criterion-related validity was not tested. Firth et al. [10] adapted the EFSI to assess stress among 725 New Zealand farmers and added a seventh factor on community issues. While a confirmatory factor analysis (CFA) supported the seven stress factors, criterion-related validity was not tested.

Third, Truchot and Andela [11] developed the 37-item Farm Stressor Inventory (FSI) based on a review of the literature and interviews with 10 farmers. They surveyed 2,142 French farmers, and an exploratory factor analysis (EFA) and CFA revealed eight factors: workload and lack of time, uncertainty about the future and the financial market, agricultural legislation pressure, social and geographical isolation, financial worry, conflicts with associates or family members, family succession of the farm, and unpredictable interference with farm work. The survey also included measures of burnout and hopelessness, and the results showed that the eight stress factors were positively and significantly correlated with emotional exhaustion, cynicism, and hopelessness. While the FSI showed evidence of criterion-related validity, the outcomes were limited to two of the three burnout scales and hopelessness. Another limitation was that the EFA and CFA were conducted on the same pool of participants by splitting the sample in half, making it unclear whether the factor structure would be supported in different contexts.

More recently, a mixed-methods study of Canadian farmers identified 13 stressors through interviews with 75 farmers and industry experts [12]. The 13 stressors were organized into four themes: living in a farming bubble (e.g., public scrutiny, financial strain, family stress), constant high-stakes decision-making (e.g., machine breakdowns, time pressure), comparison to others (e.g., farm succession, pressure to succeed), and lack of control (e.g., animal diseases, government stress). These themes, along with a review of existing farmer stressor surveys, informed a 12-item stressor scale. The researchers surveyed 1,167 farmers who rated workload and time pressure (62.6%), government policies (55.9%), unpredictability (55.0%), financial strain (52%), and uncertainty about the future (50.1%) as the most stressful issues, whereas hazardous working conditions (16.2%) and geographical isolation (13.7%) were rated as the least stressful [12]. While this research provided data on Canadian farmers, the stressor scale did not include psychometric details or criterion validity.

The persistent stress inherent in farming can adversely impact the mental health of farmers [2–4, 6, 7]. For instance, a national study of Canadian farmers showed that 57.8% reported symptoms of depression, 49.2% reported symptoms of anxiety, and exhibited high burnout scores -- all surpassing general population rates [4]. Gender comparisons also revealed that women reported higher levels of depression, anxiety, and emotional exhaustion than their male counterparts, while farmers, in general, exhibited low resilience levels [4]. Resilience refers to the ability to cope well when faced with adversity and is understood as a learned process that can safeguard against occupational stressors and mental illness [13]. Anxiety and depression have been linked to unsafe work behaviors, injuries, and lost productivity in agriculture [14]. These findings underscore the urgent need for targeted interventions to understand farm stressors and address the mental health challenges faced by farmers.

Understanding the link between work stressors and mental health in farmers can be improved by applying the Job Demands-Control (JDC) model [15]. The JDC model identifies two factors influencing mental health: job demands and job control. Job demands include pressures such as time constraints and workload, while job control refers to how much workers can influence their tasks’ order, volume, and content [15]. These factors interact to create four work environment types: high strain (high demands, low control), active (high demands, high control), passive (low demands, low control), and low strain (low demands, high control). Farmers may be in these high strain work environments, which help explain why they experience poor mental health. For example, in agriculture, job demands often include busy seasonal schedules, long hours, and physical labor, exacerbated by weather and market fluctuations. Job control may include farm size, available resources such as manpower, equipment, and navigating policy changes. Moreover, the JDC model can also be used to ensure that a survey for measuring farm stressors include items that capture a range of job demands and job control elements, supporting the content and face validity. Thus, using the JDC model for measuring farm stressors can provide a nuanced understanding of the specific pressures farmers face and their relationship to mental health which can allow for more targeted interventions.

The existing farm stress scales have several limitations including untested or limited criterion validity [8–12], non-validated outcome measures [8], do not report the scale’s factor structure [12], none have been validated for the Canadian context, and have not been grounded in a theoretical framework. Also, several scales date back to the 1990s [8, 9]. We also observed an increase in the number of stress factors measured over time, contrary to the recommendation that assessment tools provide a parsimonious analysis and interpretation [16]. Therefore, our study aimed to: (a) investigate the types of stressors experienced by farmers, (b) develop a farmer stress assessment tool and test its factor structure, internal consistency, and (c) assess its criterion-related validity to self-reported levels of anxiety, depression, burnout, and resilience among farmers.

## Methods

### Research design

We conducted a cross-sectional study whereby participants were invited to complete an online survey via SurveyMonkey. This study was part of a larger research program investigating farmer mental health in Alberta, Canada.

### Procedure

Using convenience sampling, we recruited participants from February 1 to July 31, 2023, to complete the survey online. We promoted our study on our research website and through agricultural organizations that featured the study in their newsletters and social media. As an incentive, participants could submit their email addresses on a separate SurveyMonkey survey to enter a draw to win one of three $200 prizes. Email addresses were not connected to the survey responses, and we did not collect identifying information in the survey. All participants provided their informed consent before beginning the survey by clicking an “I consent” button. This study was approved by the University of Alberta’s ethics review board (Pro00126276).

### Participants

To be included in our study, participants needed to be over 18 years old, able to read and write English, and self-identify as a farmer in any commodity. Overall, we collected data from 492 participants, and the sample was separated into two groups. Sample 1 included 354 farmers from Alberta, Canada, who were on average 40.04 years old (*SD* = 12.73), with an age range of 18 to 82 years. Nearly half were men (*n* = 164, 46.3%), and the majority were married or in a committed relationship (*n* = 303, 85.6%) and the owner/operator of their farm (*n* = 243, 68.7%). Most indicated their primary commodity was beef farming (*n* = 98, 27.7%) or oilseed/grain farming (*n* = 97, 27.4%), which are the two most common commodities produced in that province. Participants in Sample 1 completed a questionnaire containing the 20-item farmer stress survey and four validated measures assessing depression, anxiety, burnout, and resilience. Sample 2 included 138 farmers who lived outside of Alberta, Canada, who were on average 37.20 years old (*SD* = 9.97) with an age range of 18 to 86 years. This sample included more men (*n* = 86, 62.3%), and the majority were married or in a committed relationship (*n* = 111, 80.4%) and the owner/operator of their farm (*n* = 84, 60.9%). Most indicated their primary commodity was dairy farming (*n* = 43, 31.2%). Participants in Sample 2 completed the farm stress survey to assess its factor structure using CFA.

**Table 1:** Characteristics of the samples

Characteristic	Sample 1, (*N* = 354) *n* (%)	Sample 2 (*N* = 138) *n* (%)
Gender Men Women Not reported	164 (46.3%) 187 (52.8%) 3 (0.8%)	84 (60.9%) 38 (27.5%) 0 (0%)
Relationship status Married/in committed relationship Single/never married Separated/divorced Widowed	303 (85.6%) 35 (9.9%) 9 (2.5%) 5 (1.4%)	111 (80.4%) 22 (15.9%) 3 (2.2%) 2 (1.4%)
Commodity type Beef cattle Beekeeping Dairy cattle Goats (meat and dairy combined) Horticulture Oilseed and grain Swine Poultry (chicken, turkey, ducks) Sheep (meat and dairy combined) Other (elk, bison, yak, equine)	98 (27.7%) 13 (3.7%) 26 (7.3%) 20 (5.6%) 18 (5.1%) 97 (27.4%) 16 (4.5%) 36 (10.2%) 20 (5.6%) 10 (2.8%)	28 (20.3%) 2 (1.4%) 43 (31.2%) 7 (5.1%) 2 (1.4%) 17 (12.3%) 10 (7.2%) 16 (11.5%) 8 (5.8%) 5 (3.6%)

## Materials

### Background questions

The survey included three questions about their personal demographics (e.g., age, gender, relationship status), and three questions about their farm (e.g., type of farm, their role on the farm, primary commodity).

### Farmer stress assessment tool

We developed a 20-item survey tool based on a review of the literature, examining existing farm stress surveys [8–12], theory [15] and consulting 10 farmers and agricultural industry experts to learn more about the stressors they experience. The generated items were then reviewed by five farmers and two agricultural industry experts to ensure the items were clearly worded and had face and content validity. Participants were asked to rate the extent to which they found each item a source of worry or concern in the past two weeks. The two-week framework was chosen to be consistent with the scoring of the mental health assessments and because research shows shorter recall periods tend to improve the accuracy of responses [17]. Participants indicated their response using a 5-point scale with response options of 0 (not at all), 1 (very little), 2 (sometimes), 3 (quite a bit), and 4 (a great deal).

### Criterion-related validity measures

We used four validated scales to investigate mental health outcomes: anxiety, depression, anxiety, burnout, and resilience. We used one additional single item to assess mental well-being.

### Depression

The Patient Health Questionnaire (PHQ-9) was used to assess self-reported depression symptoms that occurred in the past two weeks [18]. Participants responded to each item on a four-point scale with response options ranging from 0 (not at all), 1 (several days), 2 (over half the days) and 3 (nearly every day). Responses were summed to create a total score of 0 to 27. The following cut-off scores were used to create classification categories: 0 to 4 = none/minimal, 5 to 9 mild, 10 to 14 = moderate, 15 to 21 = moderately severe and 21 to 27 = severe depression [18]. We also included a single item to assess mental well-being: *In general*,* how is your mental health?* This item was used to validate the four mental health scales and was intended to measure the participant’s overall sense of mental wellness at the time of the survey. Participants responded to this item using a 5-point scale from (1) poor to (5) excellent.

### Anxiety

The Generalized Anxiety Disorder-7 item scale (GAD-7) was used to identify self-reported symptoms of anxiety that occurred in the past two weeks [19]. The GAD-7 is a commonly used survey for anxiety in population-based studies, demonstrates good reliability, and shows cross-cultural validity [19]. The survey included seven statements, and participants responded to each on a four-point scale with response options ranging from 0 (not at all), 1 (several days), 2 (over half the days) and 3 (nearly every day). Responses were summed to create a total score of 0 to 21, The following cut-off scores were used to create anxiety classification categories: 0 to 4 = none, 5 to 9 = mild, 10 to 14 = moderate, and 15 to 21 = severe anxiety [19].

### Burnout

The Maslach Burnout Inventory-General Survey (MBI) was used to measure the three components of burnout: emotional exhaustion, cynicism, and professional efficacy [20]. The survey shows good validity and reliability and has been used previously with Canadian farmers [3]. The survey consisted of 16 items, and participants responded to each statement using a 7-point scale with response options ranging from 0 (never) to 6 (every day). Responses for each subscale are scored separately using means. A higher mean score for each subscale indicated higher emotional exhaustion, cynicism, or professional efficacy.

### Resilience

The Connor Davidson Resilience Scale (CD-RISC) 10-item version was used to assess resilience. The questionnaire defines resilience as hardiness and persistence [21]. The CD-RISC is a commonly used tool to measure resilience with farmers in Canada and demonstrated good reliability [3]. Participants were asked to rate their agreement with 10 statements in the past month using a 5-point scale with response options ranging from 0 (not true at all) to 4 (true nearly all the time). Total scores were summed, and higher scores reflected greater resilience.

### Statistical analyses

We used descriptive statistics (e.g., means, standard deviations, percentages) to examine demographic data. Participants’ scores on the four validated surveys (i.e., GAD-7, PHQ-9, CD-RISC, and MBI) were calculated according to the manuals’ specifications. Additional file 1 presents *t*-tests and analysis of variance (ANOVA) comparing farmers in Alberta (Sample 1) to Canadian farmers [4] and general population scores reported by Mental Health Research Canada in 2021 [22]. We used SPSS for all analyses. Prior to analysis, data were screened for missing values, outliers, and normality. Listwise deletion was used for missing data in the analysis involving Sample 1 because it was a large dataset, while mean substitution was used for the CFA to retain the sample size since the missing data appeared random. All tests were two-tailed, and the significance level was set at α = 0.05.

For the FSAT, we first conducted an EFA using maximum likelihood extraction with varimax rotation. An EFA is a statistical method to identify underlying categories among survey items [23]. We chose varimax rotation because it minimizes cross-loadings to simplify the factor solution [23]. Four criteria were used to determine the number of factors to rotate: the a priori hypothesis that the survey was multidimensional, the scree test, the eigenvalue greater-than-one rule, and the interpretability of the solution [24]. Survey items were retained if their factor loading was ≥ 0.30 and did not cross-load with more than one factor at the 0.30 level.

Next, we conducted a CFA to evaluate the identified factor structure of the FSAT. CFA allows researchers to specify the number of factors and the pattern of factor loadings, providing a statistical test to validate the factor structure derived from the EFA [24]. Additionally, CFA can help fine-tune the model by allowing researchers to test different model specifications, such as adding or removing items or allowing for correlated errors [24]. We conducted a CFA using the maximum likelihood procedure with SPSS Amos 29 [25]. As the χ2 values can be sensitive to sample size and often significant in models with larger samples [26], we assessed model fit using several other indices that are less sensitive to sample size including the ratio of the Chi-square statistic to the degrees of freedom (χ^2^/df), the Comparative Fit Index (CFI), the Tucker–Lewis Index (TLI), and the root mean square error of approximation (RMSEA). Following guidelines, a RMSEA ≤ 0.08, a χ^2^/df score of five or less, and a CFI and TLI score of 0.90 or higher indicate a reasonable fit of the model [24, 26].

We assessed internal consistency of each factor using Cronbach’s alpha (α) and McDonald’s omega (ω). While Cronbach’s α is a widely used measure of internal consistency, McDonald’s ω, which is based on a factor analytic approach and the correlation between items, has been shown to be more robust [27]. Unlike Cronbach’s α, McDonald’s ω remains reliable even when the assumptions of normally distributed items, equal variances, and equal factor loadings are violated [27]. Both α and ω values can range between 0 and 1, and values of 0.70 or greater are considered acceptable [28] although several researchers have noted that Cronbach’s α values of 0.65 or greater are considered adequate for social science research [29, 30].

## Results

### Types of farm stressors

**Fig. 1:**
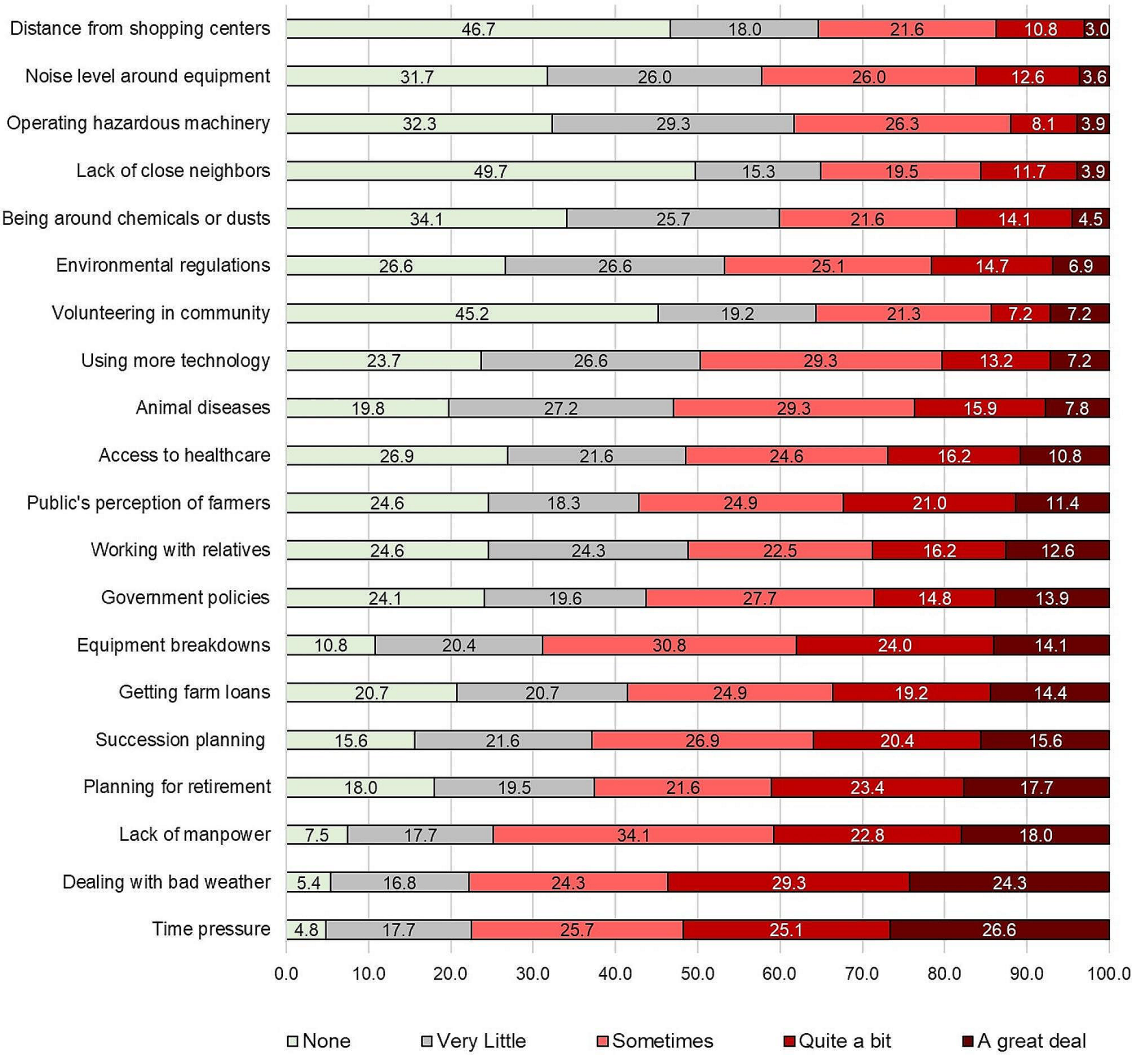
Ratings of the 20 farm stressors by participants (Sample 1, *N* = 354)

The proportions of farmers who rated their levels of stress for the 20 items are presented in Fig. 1. As shown, 13 items were rated as stressful “sometimes” to “a great deal” in the past two weeks by over 50% of the sample. The items rated most stressful included dealing with bad weather (77.8%), time pressure (77.5%), lack of manpower (74.9%), equipment breakdowns (68.9%), succession planning (62.9%), planning for retirement (62.6%), getting farm loans (58.6%), public scrutiny (57.2%), and government policies (56.3%). Comparatively, farmers rated lack of close neighbors (65%), distance to shopping (64.7%), volunteering in the community (64.4%), and operating machinery (61.7%) as causing them “very little” stress or were “not at all” stressful.

### Exploratory factor analysis

The EFA revealed that the stress survey had five underlying factors. Inspecting the factor loadings showed that four items cross-loaded onto two factors. We removed these four items and re-ran the EFA with 16 items (Table 2). The resulting solution again showed a five-factor structure that accounted for 61.60% of the variance.

**Table 2:** Factor loadings, communalities and descriptive statistics for the Farmer Stress Assessment Tool (FSAT) (sample 1, *N* = 332)

	Factor loadings					Communalities	M (SD)
	1	2	3	4	5		
**Factor 1: Unexpected work disruptions**							**2.34 (0.87)**
Equipment breakdowns	*0.56*	0.21	0.00	0.10	0.18	0.40	2.10 (1.20)
Not having enough manpower	*0.65*	0.03	0.20	0.11	0.03	0.47	2.26 (1.17)
Dealing with bad weather	*0.47*	0.07	0.18	0.00	0.09	0.30	2.50 (1.18)
Having too much to do	*0.71*	0.02	0.13	0.08	0.06	0.53	2.51 (1.19)
**Factor 2: Agricultural hazards**							**1.27 (0.94)**
Operating hazardous machinery	0.18	*0.73*	0.17	0.19	0.02	0.64	1.22 (1.10)
Being around chemicals or dusts	0.06	*0.72*	0.00	0.06	0.13	0.56	1.29 (1.20)
Noise level around equipment	0.08	*0.57*	0.01	0.26	0.28	0.48	1.30 (1.15)
**Factor 3: Farm and financial planning**							**1.96 (1.05)**
Having to get farm loans	0.14	0.10	*0.57*	0.07	0.05	0.38	1.86 (1.34)
Planning for retirement	0.24	0.03	*0.69*	0.06	0.07	0.54	2.03 (1.36)
Succession planning of the farm	0.29	0.02	*0.55*	0.17	0.22	0.46	1.99 (1.29)
**Factor 4: Isolation**							**1.07 (0.95)**
Lack of close neighbors	0.18	0.22	0.22	*0.35*	0.19	0.30	1.05 (1.23)
Having to volunteer in the community	0.05	0.15	0.04	*0.89*	0.19	0.85	1.12 (1.26)
Distance to shopping centers	0.05	0.27	0.26	*0.46*	0.05	0.36	1.05 (1.18)
**Factor 5: Regulations and Public Pressure**							**1.66 (0.95)**
Environmental regulations	0.02	0.23	0.12	0.19	*0.67*	0.55	1.49 (1.22)
Public’s perception of farmers	0.20	0.10	0.21	0.07	*0.42*	0.37	1.76 (1.33)
Government policies	0.02	0.03	0.24	0.11	*0.35*	0.33	1.75 (1.34)

We reviewed the items on each factor for conceptual clarity, applied feedback from our panel of farmers and agricultural industry experts to more fully understand each item, and assigned each factor a label. Factor 1 included items about unexpected equipment breakdowns, facing time pressure, lacking manpower to complete the work, and dealing with volatile and unpredictable weather. These breakdowns are recalled most often during busy seasons, or the equipment has become so specialized that it is difficult or expensive to find the part. Lacking manpower often reflected finding people who wanted to work long hours or engage in seasonal work. We labeled factor 1 *Unexpected work disruptions*.

Factor 2 referred to work-related risks and hazards, and included items about handling hazardous chemicals and machinery. Our panel of experts shared that agricultural farm work involves risks to one’s physical safety such as operating large machinery, driving slow-moving tractors on roads, climbing tall ladders to check grain bins, working with livestock, and handling chemicals such as pesticides. We labeled factor 2 *Agricultural hazards*.

Factor 3 consisted of items about getting financial loans, planning retirement, and succession planning. Each of these items entail a financial component about how to ensure the farm can continue to be productive, and may necessitate that the farmer forecast their future needs with limited information. Moreover, items about retirement and succession planning may point towards issues of personal identity and ensuring the family farm is passed to the next generation without disrupting family dynamics. We labeled factor 3 *Farm and financial planning.*

Factor 4 included items about having few neighbors, a limited social network, volunteering in the community, and being far from shopping centers. For many farmers who live in rural areas, there may be a certain degree of isolation expected. Several farmers commented that internet connections were often weak and unreliable in rural areas, the heavy workload or type of commodity (e.g., dairy farming) often kept them busy and limited who and how often they communicated with people outside of family members and colleagues. They also noted that rural communities in general seemed to be decreasing in size with limited resources, meaning that shopping trips were limited or required advanced planning. We labeled factor 4 *Isolation*.

Factor 5 included items related to government policies, regulations, and the public’s perception of farming, These items appeared to reflect frustration about a perceived lack of understanding about farming from people unfamiliar with agriculture. Examples included a perceived public scrutiny about farming, the treatment of animals and the environment, and changing government policies and regulations that some felt included limited consultation with farmers. We labeled factor 5 *Regulations and public pressure*. As we are testing an assessment tool, we refer to the factors as subscales.

### Psychometric properties and validity of the FSAT

The psychometric results for the five farmer stress subscales are summarized in Table 3. The internal consistencies for each scale, measured by Cronbach’s α and McDonald’s ω, ranged from 0.66 to 0.76, which are values within the acceptable range [28–30]. Because one subscale had four items and the remaining had three items each, subscale totals were calculated as mean scores to ease interpretation and allow direct comparisons. Correlations among the subscales were positive and significantly correlated, with values ranging from small to moderate.

**Table 3:** Psychometric properties and correlations among the scales of the Farmer Stress Assessment Tool (FSAT) subscales (sample 1, *N* = 332)

	Unexpected work disruptions	Agricultural hazards	Farm and financial planning	Isolation	Regulations and Public Pressure
Number of items in scale	4	3	3	3	3
Cronbach’s α	0.72	0.75	0.70	0.66	0.67
McDonald’s ω	0.71	0.76	0.71	0.67	0.67
Total variance of scale (%)	26.50%	12.64%	9.52%	6.78%	6.36%
**Correlations among scales**					
Unexpected work disruptions	--				
Agricultural hazards	0.25**	--			
Farm and financial planning	0.41**	0.21**	--		
Isolation	0.20**	0.44**	0.32**	--	
Regulations and Public Pressure	0.24**	0.32**	0.44**	0.39**	--

** *p* < .01

Examining the scale correlations shown in Table 3, we observed some evidence of discriminant and convergent validity. For example, Farm and financial planning showed a higher correlation with Regulations and public pressure (*r* = .44, *p* < .01) and Unexpected work disruptions (*r* = .41, *p* < .01), as these factors all involve external and unpredictable pressures. Likewise, Agricultural hazards and Isolation were more strongly correlated (*r* = .44, *p* < .01) as both reflected the expected realities of rural farm life. Subscales that were not expected to correlate highly, such as Unexpected work disruptions and Isolation (*r* = .20, *p* < .01), showed weaker correlations.

### Criterion-related validity of the FSAT

Table 4 shows the correlations between the FSAT subscales and the levels of depression, anxiety, burnout, resilience, and mental well-being. We examined differences by gender and commodity type, presenting only the significant findings.

### Farm stressors and Mental Health outcomes

The descriptive statistics (means, standard deviations) of the mental health outcomes for Sample 1, as well as *t* test results comparing Alberta farmers to the national sample of Canadian farmers [5] and the Canadian population norms [31] are presented in Additional file 1. For assessing criterion-related validity of the FSAT, the five farm stress subscales were significantly related to depression and anxiety. In Table 4, all five farm stressors were positively associated with depression (*r*s ranged from 0.13 to 0.40, *p*s < 0.05), but only Unexpected work disruptions (*r* = .39), Farm and financial planning (*r* = .33), and Isolation (*r* = .15) were positively associated with anxiety. Likewise, Unexpected work disruptions, Farm and financial planning, and Isolation were positively associated with emotional exhaustion and cynicism (all *p*s < 0.05). Agricultural hazards (*r* = − .11), Farm and financial planning (*r* = − .13), and Isolation (*r* = − .38) were negatively associated with professional efficacy, yet high levels of Unexpected work disruptions was associated with a high professional efficacy score (*r* = .21) suggesting that farmers persevere through hardships. Low levels of resilience were associated with high levels of Farm and financial planning (*r* = − .21), and Isolation (*r* = − .25). Mental well-being was also negatively associated with Unexpected work disruptions (*r* = − .30) and Farm and financial planning (*r* = − .26), but positively associated with Agricultural hazards (*r* = .15). We also examined item 9 of the PHQ (i.e., *Thoughts that you would be better off dead or of hurting yourself in some way*) to assess thoughts of self-harm, and found positive correlations on four of the five subscales that ranged from small to moderate (*r*’s ranged from 0.15 to 0.38).

**Table 4:** Bivariate correlations between the FSAT subscales and the mental health outcomes

	FSAT Subscales					
Mental health outcomes	Unexpected work disruptions	Agricultural hazards	Farm and Financial Planning	Isolation	Regulations and public pressure	
Depression	0.34**	0.13*	0.40**	0.38**	0.21**	
Anxiety	0.39**	− 0.03	0.33**	0.15**	0.06	
Resilience	− 0.01	− 0.02	− 0.21**	− 0.25**	− 0.02	
Emotional exhaustion	0.49**	0.04	0.36**	0.11*	0.05	
Cynicism	0.39**	0.11	0.40**	0.28**	0.18**	
Professional efficacy	0.21**	− 0.11*	− 0.13*	− 0.38**	− 0.04	
Mental well-being	− 0.30**	0.15**	− 0.26**	− 0.01	0.04	
Thoughts of self-harm	0.08	0.21**	0.26**	0.38**	0.15**	

** *p* < .01 * *p* < .05.

### Farm stressors, Well-Being, and gender

For men, higher levels of mental well-being were related to lower levels of Unexpected work disruptions (*r* = − .33, *p* < .05) and Farm and financial planning (*r* = − .23, *p* < .05). A similar pattern was found for women (*r* = − .22, *p* < .05 and *r* = − .28, *p* < .05, respectively) with exception to a positive correlation between perceived mental well-being and Agricultural hazards (*r* = .22, *p* < .05). Next, an independent *t* test examined potential gender differences for each type of stress category, and the results revealed a significant gender difference for Unexpected work disruptions, *t*(329) = -2.82, *p* < .05, with women reporting a higher level of worry (*M* = 2.47, *SD* = 0.82) than men (*M* = 2.20, *SD* = 0.82). There were no other differences for the other four types of stressors.

### Farm stressors and commodity

We conducted an ANOVA to examine potential differences in the stress categories across the commodity groups. We found significant differences for only Unexpected work disruptions, *F*(8, 315) = 2.61, *p* < .01 and Isolation, *F*(8, 315) = 5.33, *p* < .01. Post-hoc comparisons using a Bonferroni correction showed that beef farmers reported significantly higher levels of worry (*M* = 2.55, *SD* = 0.83) compared to poultry farmers (*M* = 1.93, *SD* = 0.88) for Unexpected work disruptions. For Isolation, dairy farmers reported significantly higher levels of worry (*M* = 1.89, *SD* = 0.90) compared to beef farmers (*M* = 0.87, *SD* = 0.80), grain farmers (*M* = 0.93, *SD* = 0.84), horticulture (*M* = 0.86, *SD* = 0.94) and beekeepers (*M* = 0.61, *SD* = 1.14). Likewise, Isolation stress was rated higher for pig (*M* = 1.69, *SD* = 0.87) and poultry (*M* = 1.45, *SD* = 1.06) farmers compared to beef farmers.

### Confirmatory factor analysis

**Fig. 2:**
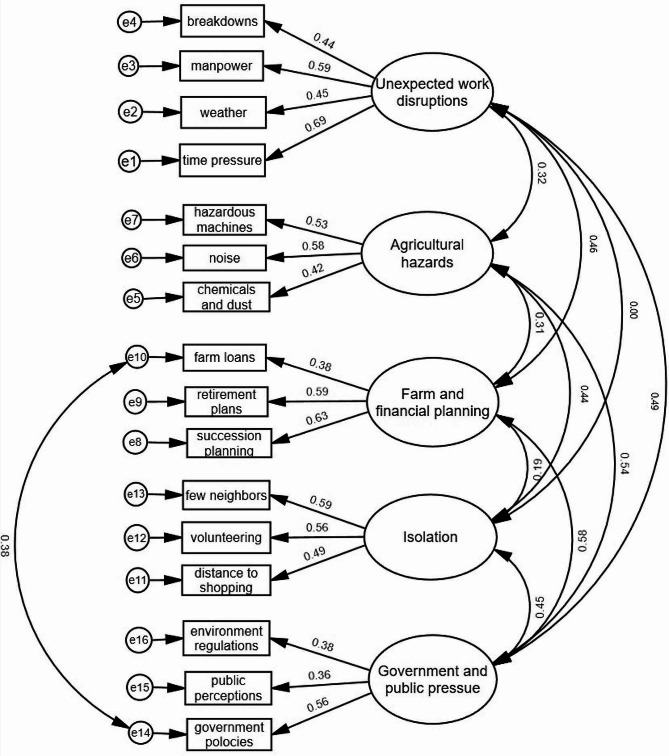
Confirmatory factor analysis (CFA) model of the FSAT (Sample 2, *N* = 138)

We tested the five-factors model using CFA using Sample 2. Although the *χ*^2^ test was significant, *χ*^2^ (94) = 138.09, *p* < .001, examination of the other fit indices that are not as sensitive to sample size revealed that the model fit was satisfactory: *χ*^2^/*df* = 1.47, RMSEA = 0.06, TLI = 0.84, and CFI = 0.87. We reviewed the modification indices, which showed that fit could be improved by correlating the error terms of item 10 (getting farm loans) and item (government policies). We reasoned that this was an appropriate modification as both items have some codependency. We re-ran the model with this single modification and found that the solution improved although the *χ*^2^ test remained significant: *χ*^2^ (93) = 123.19, *p* < .05; *χ*^2^/*df* = 1.33, RMSEA = 0.05, TLI = 0.89, and CFI = 0.91. The final model with standardized coefficients is presented in Fig. 2.

## Discussion

Consistent with previous research [8–12], our study identified that farmers experienced a wide range of occupational and personal stressors. The stressors rated most worrisome in the past two weeks for farmers in our study included adverse weather conditions, workload and time pressure, lack of manpower, equipment breakdowns, and thinking about retirement and succession planning. Using EFA, we reduced the long list of individual stressors to five categories, namely Unexpected work disruptions, Agricultural hazards, Farm and financial planning, Isolation, and Regulations and public pressure. Not only did these categories of stressors improve communication to help target specific topics to address in policies and interventions, but they also offered insights into the multifaceted nature of farmers’ lives.

Our findings are consistent with previous research that consistently reported time pressure, weather, succession planning, uncontrollable events, finances, and government policies as particularly stressful for farmers across various regions [8–12]. While these studies also reported geographical and social isolation, occupational hazards, and working with relatives as stressors, these did not emerge as overly worrisome for farmers in our study. In fact, when we reflected on the five farm stress subscales identified in our analysis, those rated most stressful tended to have an unpredictable and uncontrollable quality. For example, a farmer cannot predict or control the weather, just as they cannot predict changing policies, market prices, or when their equipment might breakdown. In contrast, a farmer living in a rural area might expect some degree of isolation, working with relatives if they operate a family farm, and must operate machinery or use products (e.g., pesticides) that involve an inherent element of risk. For instance, isolation was rated as a stressor by less than 5% of farmers in Canada [12].

However, stressors appear to vary by personal characteristics and commodity. For example, we found that women rated Unexpected work disruptions more stressful than men, and no other gender differences among the farm stress subscales emerged. This somewhat supports previous research that reported men experienced more stress related to environmental and economic conditions than women, while women tended to have slightly higher levels of geographic isolation stress than men [12, 31]. These variations in stress scores highlight previous calls to acknowledge the diversity within the farming population and the need to tailor mental health approaches [2, 32, 33]. Women have tended to report higher stress scores than men, possibly due to additional roles they often assume on family farms, such as childcare, eldercare, and supplementary healthcare employment [34].

Using EFA and CFA, we found that the FSAT was a psychometrically sound survey tool to assess farm stressors. Moreover, the subscales demonstrated good criterion-related validity to several commonly-used mental health outcomes. For example, certain farm stressors, particularly Unexpected work disruptions and Farm and financial planning, correlated with poorer mental health. It is likely that these stressors reflect the pressure and high-stakes decisions farmers regularly faced. For instance, Thompson et al. [4] reported that the stress associated with constant, high-stakes decision-making due to unpredictable markets and evolving government policies often made farmers feel overwhelmed by the significant financial decisions they had to make without clear solutions. Indeed, our findings support the interactive nature of the job demands and job control described in the JDC model [15] to better understand the nature of farm stressors and farmer mental health. While the JDC model was reviewed during the survey development to ensure our survey items had content and face validity, our findings also supported the model. For example, the stressors encompassing high psychological demands (e.g., time pressure, planning for retirement, succession planning) and low control (e.g., weather, equipment breakdowns, government policies, isolation) tended to be associated with poorer mental health outcomes. Guided by this model, it is possible to categorize farming as a high strain (high demands, low control) occupation that makes farmers vulnerable to poor mental health outcomes. In fact, we found that thoughts of self-harm in the past two weeks were significantly correlated with four of the FSAT subscales.

However, not all stress was negative. We observed a positive correlation between Agricultural hazards and the mental well-being among women farmers. Reviewing the subscale’s items showed that they reflected engaging in agricultural work, which involves more risks and traditionally male-dominated tasks. While these activities were stressful for women, they may also have been interpreted as fulfilling and personally rewarding. Recent Canadian census data showed an increasing representation of women in agriculture, with 32.4% of farm operators being women in Alberta [35]. Future research should explore the evolving roles of women in agriculture and whether their increased involvement in farming and decision-making buffers the farm-related stressors and empowers them [36, 37].

### Implications

Our findings that farmers experienced a wide range of farm stressors associated with poor mental health outcomes underscore the need for interventions not just in Alberta, but for farmers worldwide. Our findings offer valuable insights for policy-makers, aiding in their understanding of how regulations and policy changes contribute to the stress experienced by farmers. It is essential to ensure that changes to policies and regulations are effectively communicated with farmers or that farmers are actively involved in the consultation process. While future research needs to examine what drives these stressors, our findings provide some insights. For example, some of the stress associated with isolation reflect the limited ways for farmers to meaningfully engage with people outside their family or main social circle. In Alberta, approximately 67% of families living in rural areas do not have access to reliable high-speed internet [38]. Improving internet access is needed, as well as ways to encourage farmers to socialize and connect through agricultural associations and extension opportunities.

However, recognizing that many farm stressors cannot be eliminated, interventions need to be provided to help farmers cope with the stress. One of the first ways to engage farmers in this is to provide mental health literacy education. Mental health literacy involves recognizing, understanding, and managing mental disorders, which is a critical tool for fostering individual and community resilience [39]. Mental health awareness campaigns and literacy programs tailored for the agricultural industry in Canada (e.g., In The Know) have proliferated since 2016 and have significantly improved individuals’ understanding of mental health topics such as depression, anxiety, and substance use. They have also enhanced their confidence in discussing mental health issues with others [40]. While it is possible that these awareness campaigns prompted farmers to become more self-aware of their mental health concerns, thus leading to more accurate mental health reporting in our survey compared to previous Canadian surveys [3, 4], mental health literacy programs in Alberta have been limited. Given the persistent stigma surrounding mental health in rural areas [41], it is imperative to make mental health awareness and education accessible to farmers, and delivered by mental health professionals who possess experience or understanding of agriculture.

Mental health interventions must focus on equipping farmers with coping strategies. Internet-delivered mental health interventions represent a promising approach that address accessibility and stigma [42]. A Canadian study implemented an online, five-lesson course providing psychoeducation and strategies for managing depression and anxiety, with weekly therapist assistance [43]. The results showed significant reductions in depression and anxiety, decreased stress and improved resilience from pre- to post-treatment [43]. Similar outcomes were observed for farmers in Scotland, with their depression scores declining over a three-month period [42].

### Limitations

While we found support for our FSAT through an EFA and a CFA, and our survey showed acceptable reliability and validity, our study included several limitations. First, the cross-sectional nature of our research design limited our findings to a single time point and prevented us from making causal inferences about stressors and mental health. Second, selection bias may have skewed results, as individuals with mental health concerns may have been inclined to participate and inflated the depression and anxiety estimates.

Third, our CFA sample size was small. A common-rule-of-thumb was that researchers conducting a CFA should aim for a sample size of ≥ 200 [44]. However, determining the sample size for CFA depends on factors such as the number of indicators to latent variables, indicator reliability, missing data patterns, absence of cross-loadings, and model complexity (26,44,45). In addition to these considerations, some researchers recommend conducting a power analysis to estimate the sample size needed to achieve maximum statistical power for a hypothesized effect size at a specified significance level [44]. For our CFA analysis, we used an online tool [46] and determined that a sample size of 173 would provide the maximum statistical power. As our actual sample size was smaller, our ability to detect differences was reduced. Barker et al. [47] likened statistical power to the precision of a microscope: with low magnification, fine details are hard to detect, just as low power in a study can cause subtle effects to be missed. Furthermore, we acknowledge that the demographics between Study 1 and Study 2 differed slightly in gender differences. Thus, further research using a larger sample is needed to validate our findings.

## Conclusion

By developing a farmer stress survey through a review of research, existing assessment tools, and feedback from subject-matter experts, and then applying a combination of statistical techniques, we developed a psychometrically-sound and valid survey to assess the stresses farmers experience. In addition to this new survey, our findings underscore the concerning link between farm stress and the prevalence of depression, anxiety, and burnout among farmers. This heightened vulnerability highlights the need for targeted interventions to safeguard the mental well-being of this population. By examining a subset of key stressors, our research offers a valuable roadmap for future intervention strategies. Our findings can help policymakers, public health officials, and mental healthcare providers make informed decisions pertaining to mental health services and training opportunities.

### Electronic supplementary material

Below is the link to the electronic supplementary material.

Supplementary Material 1

## Data Availability

The datasets used and/or analyzed during the current study are available from the corresponding author on reasonable request.

## Abbreviations

FSAT: Farmer Stress Assessment Tool
EFA: Exploratory factor analysis
CFA: Confirmatory factor analysis
PHQ-9: Patient Health Questionnaire
GAD-7: Generalized Anxiety Disorder-7 item scale
MBI: Maslach Burnout Inventory-General Survey
CD-RISC: Connor Davidson Resilience Scale
