# Supplementary material for: A multidimensional tool to measure farm stressors: development and initial validation of the farmer stress assessment tool (FSAT)
Source: BMC Psychol. 2024 Aug 12;12:435. doi: 10.1186/s40359-024-01929-w (PMC11321130; doi:10.1186/s40359-024-01929-w)
Supplement: Supplementary file 1 — Supplementary Material 1 [file 40359_2024_1929_MOESM1_ESM.docx]

**Additional file 1.** Descriptive statistics (n, mean, standard deviations) for each validated scale used for the overall 2021 sample and by gender, and with comparisons to scale population norms and 2015/2016 survey data, where available.

|  | **Alberta Farmers^a^** | | | **Canadian Farmers^b^** | | | **Population Norm^c^** | | |
| --- | --- | --- | --- | --- | --- | --- | --- | --- | --- |
| **Scale** | ***n*** | ***M* (*SD*)** | ***p* value** | ***n*** | ***M* (*SD*)** | ***p* value** | ***n*** | ***M* (*SD*)** | ***p* value** |
| **Depression (PHQ-9)** | | |  |  |  |  |  |  |  |
| Total | 354 | 8.17 (5.9) | - | 978 | 5.89 (5.3) | < .001 | 5018 | 2.91 (3.5) | < .001 |
| Men | 164 | 8.64 (6.0) | 0.06 | 562 | 5.25 (4.9) | < .001 | 2326 | 2.70 (3.5) | < .001 |
| Women | 187 | 7.64 (5.7) |  | 363 | 6.87 (5.6) | 0.33 | 2692 | 3.10 (3.5) | < .001 |
|  |  |  |  |  |  |  |  |  |  |
| **Anxiety (GAD-7)** | |  |  |  |  |  |  |  |  |
| Total | 354 | 8.77 (5.2) | - | 980 | 6.12 (4.9) | < .001 | 5030 | 2.97 (3.4) | < .001 |
| Men | 164 | 8.23 (5.4) | < .05 | 563 | 5.45 (4.6) | < .001 | 2332 | 2.66 (3.2) | < .001 |
| Women | 187 | 9.22 (4.9) |  | 364 | 7.16 (5.2) | < .001 | 2698 | 3.20 (3.5) | < .001 |
|  |  |  |  |  |  |  |  |  |  |
| **Emotional Exhaustion** | | |  |  |  |  |  |  |  |
| Total | 346 | 2.98 (1.5) |  | 973 | 2.64 (1.6) | < .001 | 47,800 | 2.26 (1.5) | < .001 |
| Men | 160 | 2.69 (1.5) | < .001 | 560 | 2.46 (1.6) | 0.03 |  |  |  |
| Women | 183 | 3.23 (1.5) |  | 360 | 2.92 (1.6) | 0.003 |  |  |  |
|  |  |  |  |  |  |  |  |  |  |
| **Cynicism** |  |  |  |  |  |  |  |  |  |
| Total | 346 | 2.59 (1.4) |  | 973 | 2.20 (1.4) | < .001 | 47,752 | 1.74 (1.4) | < .001 |
| Men | 160 | 2.60 (1.4) | 0.44 | 560 | 2.16 (1.4) | < .001 |  |  |  |
| Women | 183 | 2.58 (1.4) |  | 360 | 2.26 (1.5) | < .001 |  |  |  |
|  |  |  |  |  |  |  |  |  |  |
| **Professional Efficacy** | | |  |  |  |  |  |  |  |
| Total | 346 | 4.17 (1.4) |  | 973 | 4.70 (1.1) | < .001 | 47,843 | 4.34 (1.2) | 0.013 |
| Men | 160 | 4.28 (1.4) | 0.12 | 560 | 4.80 (1.1) | < .001 |  |  |  |
| Women | 183 | 4.09 (1.5) |  | 360 | 4.69 (1.1) | < .001 |  |  |  |
|  |  |  |  |  |  |  |  |  |  |
| **Resilience (CD-RISC)** | | |  |  |  |  |  |  |  |
| Total | 352 | 25.53 (6.5) |  | 980 | 24.66 (6.2) | < .0.01 | 764 | 31.80 (5.4) | < .001 |
| Men | 164 | 26.46 (6.8) | < .01 | 562 | 25.04 (6.1) | < .0.01 | 218 | 33.53 (4.6) | < .001 |
| Women | 185 | 24.74 (6.1) |  | 365 | 24.10 (6.2) | < .0.05 | 546 | 31.08 (5.6) | < .001 |

*Note.* ^a^ Statistical comparison within our data. ^b^ Statistical comparison of our data with the 2021 Canadian Farmer data. ^c^ Statistical comparison of our data with population normative data.

** *p* < .01 * *p* < .05.
